# Ammonium tetrathiomolybdate treatment targets the copper transporter ATP7A and enhances sensitivity of breast cancer to cisplatin

**DOI:** 10.18632/oncotarget.12992

**Published:** 2016-10-31

**Authors:** Cristine L. Chisholm, Haitao Wang, Ada Hang-Heng Wong, Guelaguetza Vazquez-Ortiz, Weiping Chen, Xiaoling Xu, Chu-Xia Deng

**Affiliations:** ^1^ Faculty of Health Sciences, University of Macau, Macau SAR, China; ^2^ Genetics of Development and Disease Branch, National Institute of Diabetes, Digestive and Kidney Diseases, National Institutes of Health, Bethesda, Maryland MD, USA; ^3^ Genomics Core Facility, National Institute of Diabetes, Digestive and Kidney Diseases, National Institutes of Health, Bethesda, Maryland MD, USA

**Keywords:** cisplatin, breast cancer, ATP7A, copper, resistance, sequestering

## Abstract

Cisplatin is an effective breast cancer drug but resistance often develops over prolonged chemotherapy. Therefore, we performed a candidate approach RNAi screen in combination with cisplatin treatment to identify molecular pathways conferring survival advantages. The screen identified ATP7A as a therapeutic target. ATP7A is a copper ATPase transporter responsible for intercellular movement and sequestering of cisplatin. Pharmaceutical replacement for ATP7A by ammonium tetrathiomolybdate (TM) enhanced cisplatin treatment in breast cancer cells. Allograft and xenograft models in athymic nude mice treated with cisplatin/TM exhibited retarded tumor growth, reduced accumulation of cancer stem cells and decreased cell proliferation as compared to mono-treatment with cisplatin or TM. Cisplatin/TM treatment of cisplatin-resistant tumors reduced ATP7A protein levels, attenuated cisplatin sequestering by ATP7A, increased nuclear availability of cisplatin, and subsequently enhanced DNA damage and apoptosis. Microarray analysis of gene ontology pathways that responded uniquely to cisplatin/TM double treatment depicted changes in cell cycle regulation, specifically in the G1/S transition. These findings offer the potential to combat platinum-resistant tumors and sensitize patients to conventional breast cancer treatment by identifying and targeting the resistant tumors' unique molecular adaptations.

## INTRODUCTION

Breast cancer affects approximately one in eight women in Western countries. In the U.S., more than 200,000 new breast cancer cases are diagnosed each year, with about 5% of which is caused by mutations in breast cancer associated gene 1 (*BRCA1*) and breast cancer associated gene 2 (*BRCA2*) [1–4]. Platinum drugs such as cisplatin (*cis*-[PtCl_2_(NH_3_)_2_], *cis*-diamminedichloroplatinum(II), cDDP), carboplatin and oxaliplatin serve as conventional treatment for breast cancer and other solid tumors [5–7]. Cisplatin is a crosslink-inducing DNA-damaging agent that causes cell death primarily via adduct-formation through adjacent guanine residues [8–10]. Cisplatin may also induce cell death by damaging cytoplasmic proteins, inducing apoptosis at the execution phase level [8, 9, 11].

Platinum agents are highly effective in combating *BRCA1*-associated breast cancer because there is defect in the homology-directed DNA repair capability of these tumors that contributes to genomic instability [12, 13]. Unfortunately, resistance to platinum agents often develops, through cellular adaptations that result in reduced drug uptake, increased efflux and sequestering, and enhanced detoxification, contributing to metastasis and overall treatment failure [14, 15]. Previous studies also indicate that altered gene expression, DNA copy number changes, and substantial genomic instability contribute to cisplatin resistance [9, 15, 16]. This underscores the need for identification of alternative and ameliorative treatments that re-sensitize cells to platinum agents.

In this study, we conducted an RNAi library screening combined with cisplatin treatment in human and mouse breast cancer cell lines to identify potential therapeutic agents. The copper transporting P-type ATPase, ATP7A [17], was one of the candidates that emerged from our screen. Herein, we describe how ATP7A specifically contributes to cisplatin resistance in breast cancer, and how combining cisplatin and ammonium tetrathiomolybdate (TM), which degrades ATP7A, to sensitizes breast tumor cells to cisplatin.

## RESULTS

### Candidate RNAi screen revealed ATP7A as a target for inducing cisplatin sensitivity

To identify specific gene and pathway targets that confer to cisplatin sensitivity upon knockdown, we utilized RNAi library screen combined with cisplatin treatment in human breast cancer cell lines. This screen was carried out in three human breast cancer cell lines, MDA-MB-231, T47D and MCF7, respectively. These cell lines were determined to be resistant to cisplatin by National Cancer Institute (NCI) *In Vitro* Cell Line Screening Project (IVCLSP). Cells were treated with cisplatin alone at its IC_50_ (50% lethality) dose of 10 μM for MCF-7 and 36 μM for MDA-MB-231 and T47D, or in combination with a human siRNA siGENOME library (Thermo Dharmacon). Our RNAi library comprised of siRNA against 55 custom-selected genes (Supplementary Table S1), including genes identified in the common genomic gain regions found in the cisplatin resistant breast cancer cells and associated with poor prognosis in breast cancer, which are located on chromosomes 6p12, 6p21, 11q13, 20q13.2 and several regions of 14q [18–21]. Additionally, we included siRNA targeting genes related to stem cell maintenance, such as *SOX2* and *OCT3/4*, as well as drug detoxifying enzymes, and transporters involved in drug and metal flux.

**Figure 1:**
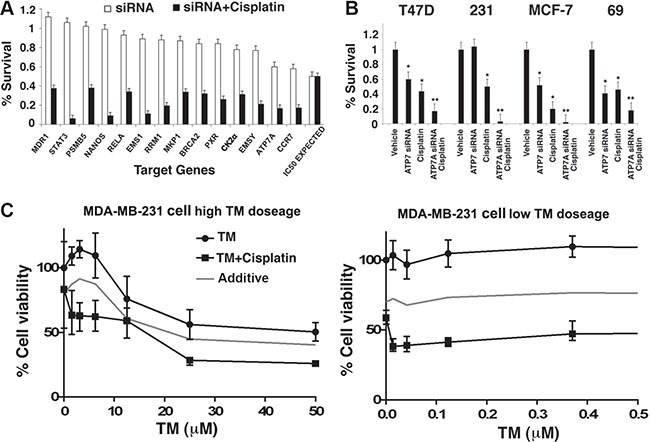
Identification and validation of a target pathway that affects cisplatin response in human breast cancer (**A**) 14 out of 55 candidate gene siRNAs that exhibited synergistic effect with cisplatin upon double treatment in T47D cells were shown. Cell survival rate was measured by ATP release assay after treatment with 36 μM (IC_50_) cisplatin with or without siRNA, and normalized to both the non-targeting siRNA and vehicle control. (**B**) Validation of synergistic effect of cisplatin and *ATP7A* knockdown in three human breast cancer cell lines T47D, MDA-MB-231 and MCF-7, and one mouse breast cancer cell line 69 using RNAi pools distinct from the initial candidate screen. (**C**) Dose response curve of MDA-MB-231 upon TM treatment in the absence or presence of 10 μM cisplatin. MTT assay was used to determine cell survival rate after 3 days drug treatment.

In our RNAi screen, 14 out of 55 (25.5%) of the candidates exhibited synergy in cell killing when combined with cisplatin at corresponding cisplatin IC_50_ dose (Figure 1A), some of which were reported to contribute to cisplatin resistance when overexpressed, such as *STAT3*, *MDR1* and *ATP7A* [22, 23]. To validate the RNAi result, the human breast cancer cell lines T47D, MDA-MB-231 and MCF7, as well as the *BRCA1*-mutant mouse breast cancer cell line 69, were treated with cisplatin alone or in combination with *ATP7A* siRNA respectively. Indeed, significantly enhanced cytotoxicity was achieved with combined treatment of *ATP7A* siRNA and cisplatin in all cell lines (Figure 1B). Increased protein levels of ATP7A or ATP7B (both are copper export pumps) were reported to correlate to cisplatin resistance a in several human cancer cell lines examined [24, 25]. Studies also showed that ATP7A sequesters cisplatin into cell vesicles (such as lysosomes) [26, 27]. Therefore, we chose to study the role of ATP7A in cisplatin resistance.

### Ammonium tetrathiomolybdate treatment sensitizes breast cancer cells to cisplatin

We first tested three metal chelating agents: neucoprine ([C_14_H_12_N_2_], 2,9-dimethyl-1,10-phenanthroline), ammonium tetrathiotungstate ([(NH_4_)_2_WS_4_], TT), and ammonium tetrathiomolybdate ([(NH_4_)_2_MoS_4_], TM). While neucoprine and TT treatment alone or in combination with cisplatin on the four cell lines under investigation did not have significant effect on cell survival, our results indicated that double treatment with TM and cisplatin significantly sensitized breast cancer cells to a level comparable to that attained with *ATP7A* siRNA (data not shown). Furthermore, we plotted a dose response curve of TM in the MB-MDA-231 human breast cancer cell line at its cisplatin IC_30_ dose of 10 μM, or in the absence of cisplatin. A 20% decrease in overall cell survival was observed after cisplatin/TM treatment as compared to the predicted additive curve (Figure 1C). It is noteworthy that the synergy between TM and cisplatin occurred at very low TM concentration, which had virtually very low or no effect on cell viability (Figure 1C).

TM is designated an orphan drug in the U.S., and was first used therapeutically in the treatment of copper toxicosis and Wilson's disease [28, 29]. TM serves as an attractive anti-cancer compound on the basis of its ability to act as both an angiogenesis inhibitor and copper trafficking protein inhibitor [30, 31], and is currently being tested in clinical trials in combination with doxorubicin and alone for the treatment of metastatic breast cancer [32]. We hypothesized that TM exerts distinct ameliorative effects in combination with conventional platinum chemotherapy independent of its effects on tumor vascularization.

### Cisplatin and TM synergistically inhibit tumor progression through inhibition of cancer stem cells accumulation and proliferation

**Figure 2:**
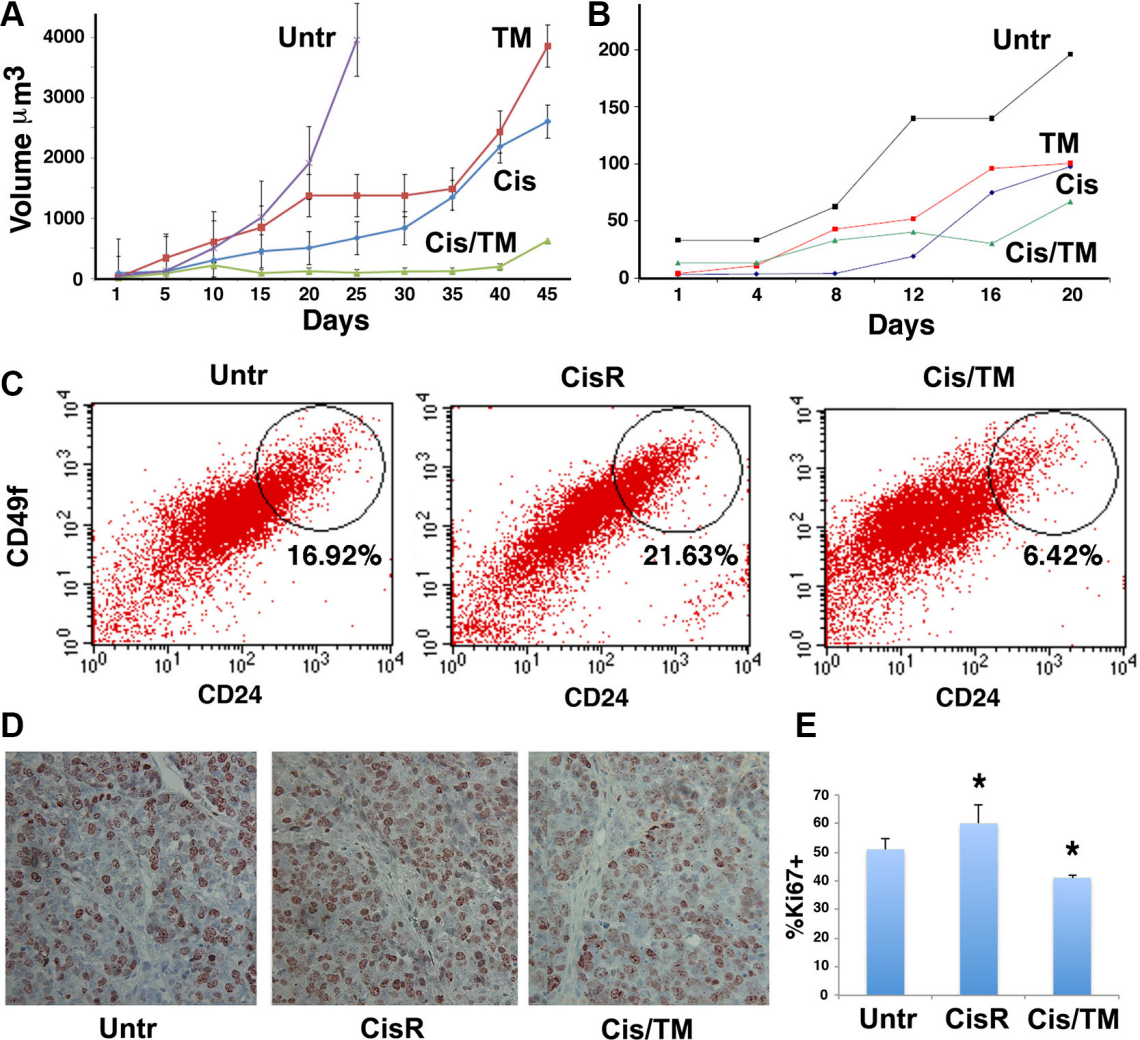
Cisplatin/TM combined therapy inhibits cancer growth and cancer stem cell accumulation *in vivo* (**A**, **B**) Average tumor volumes were measured in athymic nude mice implanted with allografts of *BRCA1*-mutant mouse 69 breast cancer cells (A) or xenografts of human MDA-MB-231 breast cancer cells (B). Mice received cisplatin (Cis), TM, cisplatin/TM (Cis/TM), or no treatment (Untr) (*N* = 5 per group). (**C**) Percentage of CD24^hi^ and CD49f^hi^ cells was profiled using combination of CD24 and CD49f antibodies. Flow cytometry analysis of 69 allograft tumor-dissociated cells revealed an elevation of CD24^hi^CD49f^hi^ CSC population in cisplatin mono-treated resistant tumors (CisR) as compared to untreated tumors (Untr), whereas a decline of CSC population was observed in cisplatin/TM double treated tumors (Cis/TM) (**D**, **E**) Ki67 positive cells were detected by immunohistochemical staining with an antibody against Ki67 (D) and shown as percentage of total cells (E). *P < 0.05 by Student's *T*-test.

Afterwards, we tested the effect of cisplatin/TM double treatment *in vivo* utilizing athymic nude mice implanted with breast cancer cells. For this experiment, 1 × 10^6^ *BRCA1*-mutant mouse breast cancer cells were implanted in the bilateral 4^th^ mammary fat pads of athymic nude mice at 6–10 weeks of age. Tumors became visible 7–14 days post-implantation, and drug administration was initiated when tumor reached 200 mm^3^. Mice were intraperitoneally (IP) injected with cisplatin at 6 mg/kg body weight with or without oral administration of TM at 0.030 mg/mL in drinking water. Tumor volume of cisplatin/TM double treated mice (Cis/TM) was significantly smaller than untreated (Untr) and mono-treated (TM or Cis) groups in 69 allograft model (Figure 2A). Mice treated with cisplatin alone had smaller tumor volume and longer survival than the mice treated with TM alone (data not shown). Similar tumor progression profile was observed in MDA-MB-231 xenograft mice (Figure 2B).

We have previously shown that prolonged treatment of cisplatin induces cisplatin resistance that is accompanied by accumulation of cancer stem cells (CSCs) [13]. This was observed in cisplatin mono-treated 69 allograft tumors harvested at 45 days post-treatment, as seen by the stark rise of tumor growth curve (Figure 2A). Thus, we refer to this tumor as the cisplatin mono-treated resistant tumor (CisR) hereafter. Flow cytometry analysis of 69 allograft tumor-dissociated cells revealed an elevation of CD24^hi^ CD49f^hi^ CSC population in cisplatin mono-treated resistant tumors (CisR) as compared to untreated tumors (Untr), whereas a decline of CSC population was observed in cisplatin/TM double treated tumors (Cis/TM) (Figure 2C).

Using these 69 allograft tumors, we analyzed cell proliferation using Ki67, a common biomarker used for the diagnosis of aggressiveness of many types of cancers, including breast cancer. Immunostaining of 69 allograft mouse tumor sections showed increased Ki67 antibody staining on untreated mouse tumor (Untr) and cisplatin mono-treated resistant tumors (CisR), as compared to cisplatin/TM double treated tumors (Cis/TM) (Figure 2D). Quantification of Ki67 staining depicted over 20% reduction in Ki67 staining in cisplatin mono-treated resistant tumors (CisR) as compared to cisplatin/TM double treated tumors (Cis/TM) (Figure 2E). Furthermore, the increase in Ki67 staining in cisplatin mono-treated resistant tumors (CisR) as compared to untreated tumors (Untr) in this experiment (Figure 2E) suggested that acquisition of cisplatin resistance correlated to increased cell proliferation. Conversely, cisplatin/TM double treated tumors had retarded tumor growth, less CSCs and lower Ki67 levels. Taken together, our allograft model suggested that TM overcomes cisplatin resistance through inhibition of cancer stem cells accumulation and cell proliferation.

**Figure 3:**
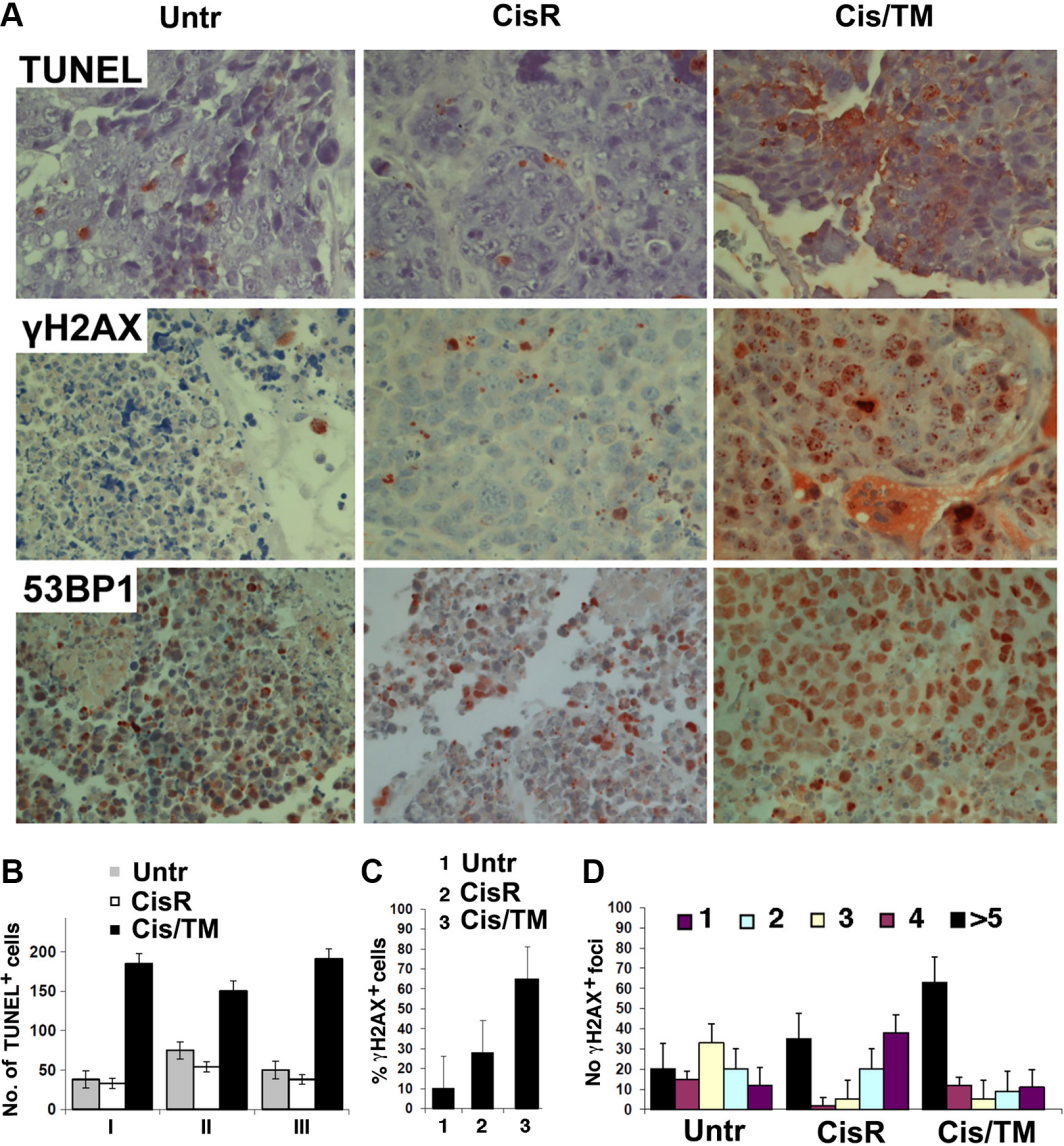
TM enhances cisplatin sensitivity in cisplatin/TM combined therapy in athymic nude mouse models through enhancement of DNA damage and apoptosis (**A**) Apoptosis and DNA damage was revealed by immunohistochemical analysis of tumor sections from allografted mice that developed resistance to cisplatin or responded to cisplatin/TM (Cis/TM) treatment with tumor regression. (**B**) Number of TUNEL^+^ cells per arbitral area in untreated (Untr), cisplatin resistant (CisR) and cisplatin/TM (Cis/TM) treated tumors. I, II and III represent three different cancers. (**C**) Number of γH2AX^+^ cells per arbitral area in untreated (Untr), cisplatin resistant (CisR) and cisplatin/TM (Cis/TM) treated tumors. (**D**) Number of γH2AX foci per nucleus in untreated (Untr), cisplatin resistant (CisR) and cisplatin/TM (Cis/TM) treated tumor cells.

Aside from CSC accumulation and cell proliferation, we also performed terminal dUTP nicked end labeling (TUNEL) assay and immunohistochemical staining of antibodies against γH2AX and 53BP1, in order to assess apoptosis through DNA fragmentation and DNA damage. Results showed that cisplatin mono-treatment resulting in resistance emergence (CisR) led to more TUNEL-positive staining and γH2AX foci as compared to untreated mouse allograft tumors (Untr), whereas further elevation was observed in cisplatin/TM double treated mouse allograft tumors (Cis/TM) (Figure 3A), suggesting that TM promotes apoptosis and DNA damage. Quantification of the stained sections showed that the number of TUNEL-positive cells (Figure 3B) in cisplatin/TM double treated tumors (Cis/TM) was positively correlated to the percentage of γH2AX-positive cells (Figure 3C) and increased γH2AX foci (Figure 3D), as compared to cisplatin mono-treated resistant tumors (CisR) and untreated tumors (Untr). Consistent with this, elevated foci of 53BP1, which is recruited to sites of aberrant fork structures to suppress homologous recombination and facilitate non-homologous end joining [33], was observed in cisplatin/TM double treated tumors (Cis/TM) as compared to cisplatin mono-treated resistant tumors (CisR) and untreated control (Untr) (Figure 3A). Taken together, these data suggested that TM enhances tumor apoptosis synergistically with cisplatin and overcomes cisplatin resistance by increasing platinum-induced DNA damage and cell death in breast cancer.

### Microarray analysis reveals changes in gene expression unique to cisplatin and TM combinatorial treatment

**Figure 4:**
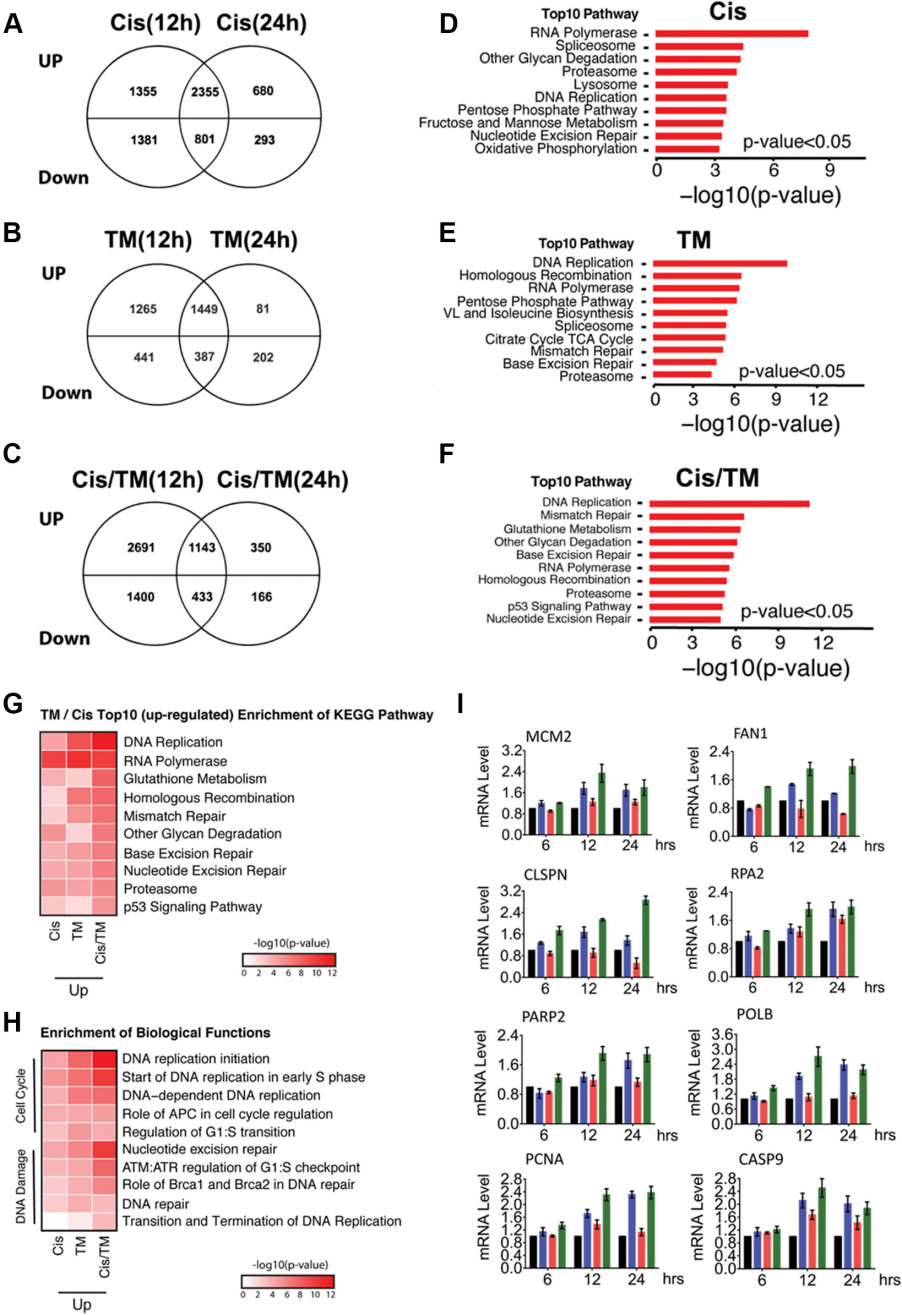
Microarray analysis of MDA-MB-231 cells treated with cisplatin (Cis), TM or cisplatin/TM (Cis/TM) (**A**–**C**) Venn diagrams depicting the number of unique upregulated or downregulated genes in cisplatin (Cis) (A), TM (B) and cisplatin/TM (Cis/TM) (C) treatment groups. (**D**–**F**) Bar graphs showing the top 10 enriched pathways by KEGG pathway enrichment analysis to demonstrate the difference in gene enrichment in cisplatin (Cis) (D), TM (E) and cisplatin/TM (Cis/TM) (F) treatment groups. (**G**) KEGG pathway enrichment heatmap to demonstrate the top10 upregulated pathway in cisplatin/TM treated cells and their changes in other treatment conditions. (**H**) GO function enrichment heatmap to highlight the enrichment of upregulated genes in the DNA Damage and Cell Cycle related functions under different treatment conditions. (**I**) Validation of gene expression by RT-qPCR.

To study the mechanism underlying synergistic action between cisplatin and TM on tumor growth, we compared changes in gene expression using microarray and conducted pathway analysis to investigate changes in gene expression profiles in MDA-MB-231 breast cancer cells under treatment with TM, cisplatin and cisplatin/TM. Our data revealed that cisplatin treatment for 12 hours and 24 hours induced expression change of 5,884 genes and 4,127 genes, respectively. TM treatment at these two time points induced expression change of 3,539 and 2,119 genes respectively, while cisplatin and TM double treatment induced expression change of 5,665 and 2,088 genes at 12 hours and 24 hours post-treatment respectively (Supplementary Table S3). Next, we used the Venn diagram to identify genes whose expression was altered at both 12 hours and 24 hours under each treatment condition. Data indicated that 3,156 genes (2,355 upregulated and 801 downregulated genes) overlapped at both time points upon cisplatin treatment (Figure 4A); 1,836 genes (1,449 upregulated and 387 downregulated genes) overlapped at both time points upon TM treatment (Figure 4B); and 1,576 genes (1,143 upregulated and 433 downregulated genes) overlapped at both time points upon cisplatin/TM double treatment (Figure 4C). Enrichment of KEGG pathway analysis of these common genes (including both upregulated and downregulated genes) identified pathways that were significantly changed in cisplatin, TM and cisplatin/TM treated tumors (Supplementary Figure S2). Inspection of the top 10 upregulated pathways under these treatment conditions identified 3 pathways in common, i.e. RNA polymerase, proteasome, and DNA replication, together with a number of pathways involved in DNA damage repair (Figure 4D–4F). Because double treatment of cisplatin and TM is most effective in triggering DNA damage, apoptosis, and inhibition of cancer growth, we first focused on the pathways changed in the double treated cells. Of note, the majority of the top 10 pathways is involved in cell cycle, DNA repair and damage response (i.e. DNA replication, RNA polymerase, homologous recombination, mismatch repair, base excision repair, nucleotide excision repair and p53 signaling), which may be accountable for the phenotypes observed in these cells (Figure 4G). Analysis of the microarray data using enrichment of biological functions highlighted changes in DNA replication, DNA damage repair, and cell cycle regulation and checkpoint (Figure 4H), which also attribute to the phenotype observed in the cisplatin/TM double treated cells. On the other hand, inspection of the top 10 downregulated pathways under cisplatin/TM treatment did not reveal such a causal relationship between drug treatment and phenotype (data not shown).

These pathways also exhibited more extensive changes (both in the number of genes and their expression levels) in the cisplatin/TM double treated cells than cisplatin or TM mono-treated cells (Figure 4G and 4H, and Supplementary Figure S3), which is consistent to the synergy between these two drugs as demonstrated in this study. Further analysis indicated that 21 genes are involved in various aspects of DNA damage (Supplementary Figure S4A), 47 in DNA damage response (DDR) (Supplementary Figure S4B), 26 in cell cycle regulation and checkpoint (Supplementary Figure S4C), and 33 in cell death (Supplementary Figure S4E). Real-time quantitative PCR (RT-qPCR) validation confirmed expression patterns of these genes (Figure 4I). Thus, cisplatin and TM double treatment significantly enhances DNA damage, activates DDR and multiple cell cycle checkpoints, leading to cell cycle arrest allowing cells to repair DNA damage, or cell death if the DNA damage cannot be repaired. Taken together, the result of microarray analysis is consistent with our observation in mouse allograft tumor.

### TM promotes cisplatin localization in cell nucleus

Next, we sought to understand the mechanism of how TM enhances DNA damage in breast cancer. It was reported that TM inhibits tumor growth through its role in copper chelation and angiogenesis inhibition [30, 31, 34, 35]. We did not see an obvious connection between angiogenesis inhibition and the markedly enhanced DNA damage in the cisplatin/TM double treated cells. Instead, we believed this might be associated with nuclear availability of cisplatin upon combined treatment with TM.

To investigate this, we engrafted *BRCA1*-mutant 69 mouse breast cancer cells in athymic nude mice. Mono-treatment with cisplatin alone resulted in cisplatin resistant tumors that grew rapidly to 3,000 mm^3^ in 32 days, while cisplatin/TM double treated tumors exhibited retarded growth at 1,000 mm^3^ 60 days post-treatment (data not shown). Freshly isolated cisplatin mono-treated resistant (CisR) and cisplatin/TM double treated (Cis/TM) mouse allograft mammary tumors were subject to comparative analyses by ImageStream multi-spectral imaging flow cytometry.

**Figure 5:**
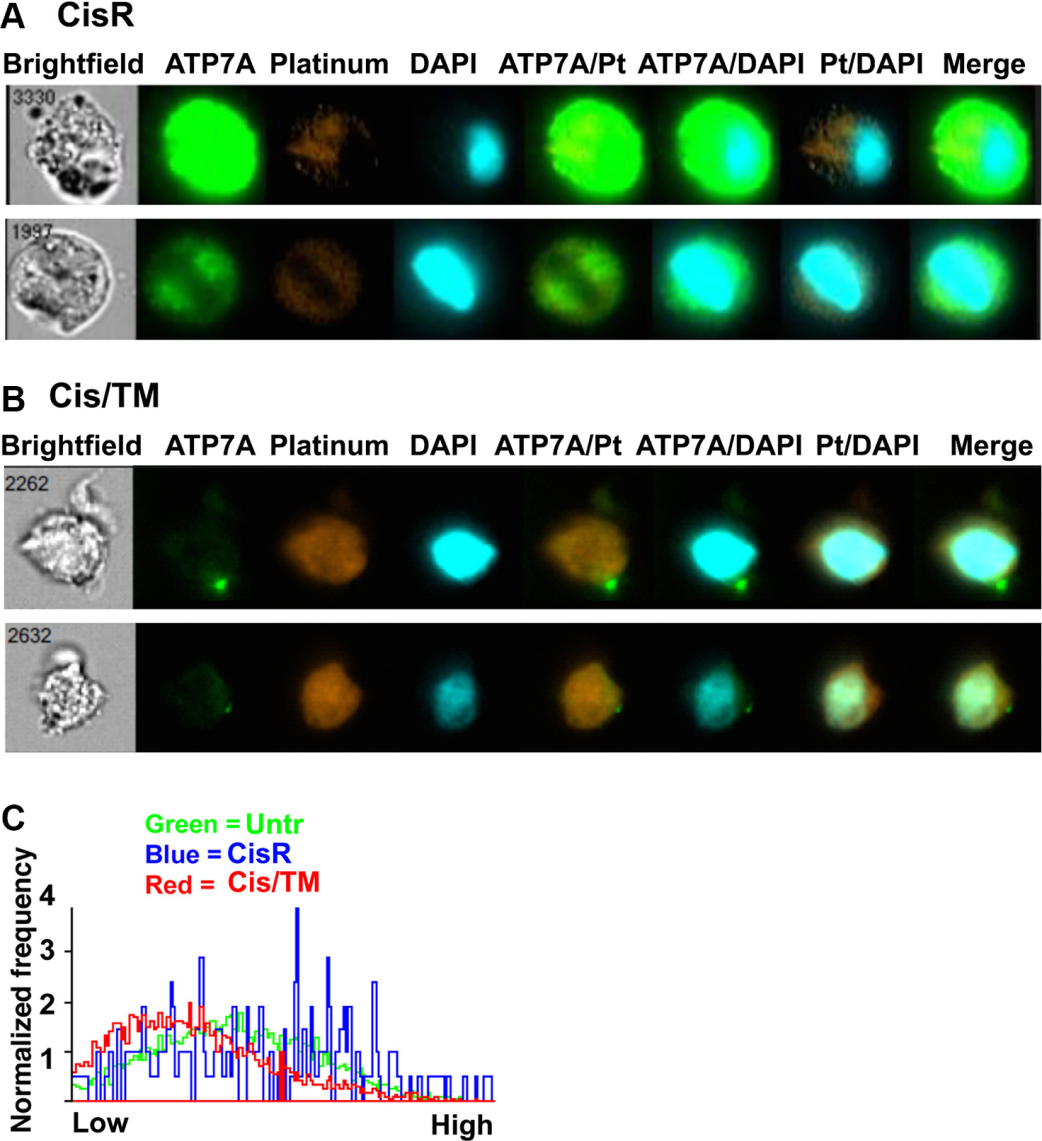
Co-localization between ATP7A, cisplatin and cell nucleus (**A**, **B**) ImageStream analysis of co-localization between ATP7A, cisplatin (Pt) and cell nucleus (DAPI) in mouse 69 allograft breast cancer cells. (**C**) Frequency plot of similarity scores to demonstrate ATP7A and cisplatin co-localization in untreated, cisplatin resistant and cisplatin/TM treated mouse 69 allograft breast cancer cells after ImageStream analysis. X-axis shows arbitrary similarity scale from ”low” (lower degree of co-localization) to ”high” (greater degree of co-localization) between ATP7A and cisplatin. Y-axis is normalized frequency reflecting the quantity of cells scored. TM treatment significantly reduced the co-localization between ATP7A and cisplatin. Over 5,000 cells were analyzed for each group of sample.

We observed key differences between cisplatin localization, and ATP7A protein expression and distribution in the mammary tumor cells. In cisplatin resistant cells (CisR), ATP7A expression was high, while cisplatin intensity (Pt) is low and is mainly excluded from the nucleus (Figure 5A). In contrast, cells isolated from cisplatin/TM double treated mammary tumors had significantly lower amounts of ATP7A and the protein was frequently observed to be localized in distinct, punctuate regions of the cell (Figure 5B). Coherently, the amount of cisplatin co-localization with nuclear DNA (DAPI) in cisplatin/TM doubled treated cells (Cis/TM) is significantly greater (Figure 5B) than that observed in cisplatin resistant cells (CisR) (Figure 5A).

Co-localization between ATP7A and cisplatin was further analyzed and plotted as normalized frequency against an arbitrary similarity scale from ”low” to ”high” using ImageStream's Quantitative Mean Similarity Score. The results showed that the cisplatin resistant tumor cells (CisR) exhibited extensive co-localization between cisplatin and ATP7A in the cytoplasm (Figure 5C), suggesting that ATP7A sequesters cisplatin and prevents it from getting into the nucleus. Cisplatin/TM double treated cells (Cis/TM) prevailed the lowest co-localization score, while untreated cells (Untr) displayed intermediate levels of cisplatin and ATP7A localization (Figure 5C). This observation supports the notion that ATP7A prevents the nuclear localization of cisplatin by sequestering and pumping it out of cells.

### TM reduces ATP7A protein level but not gene transcription

**Figure 6:**
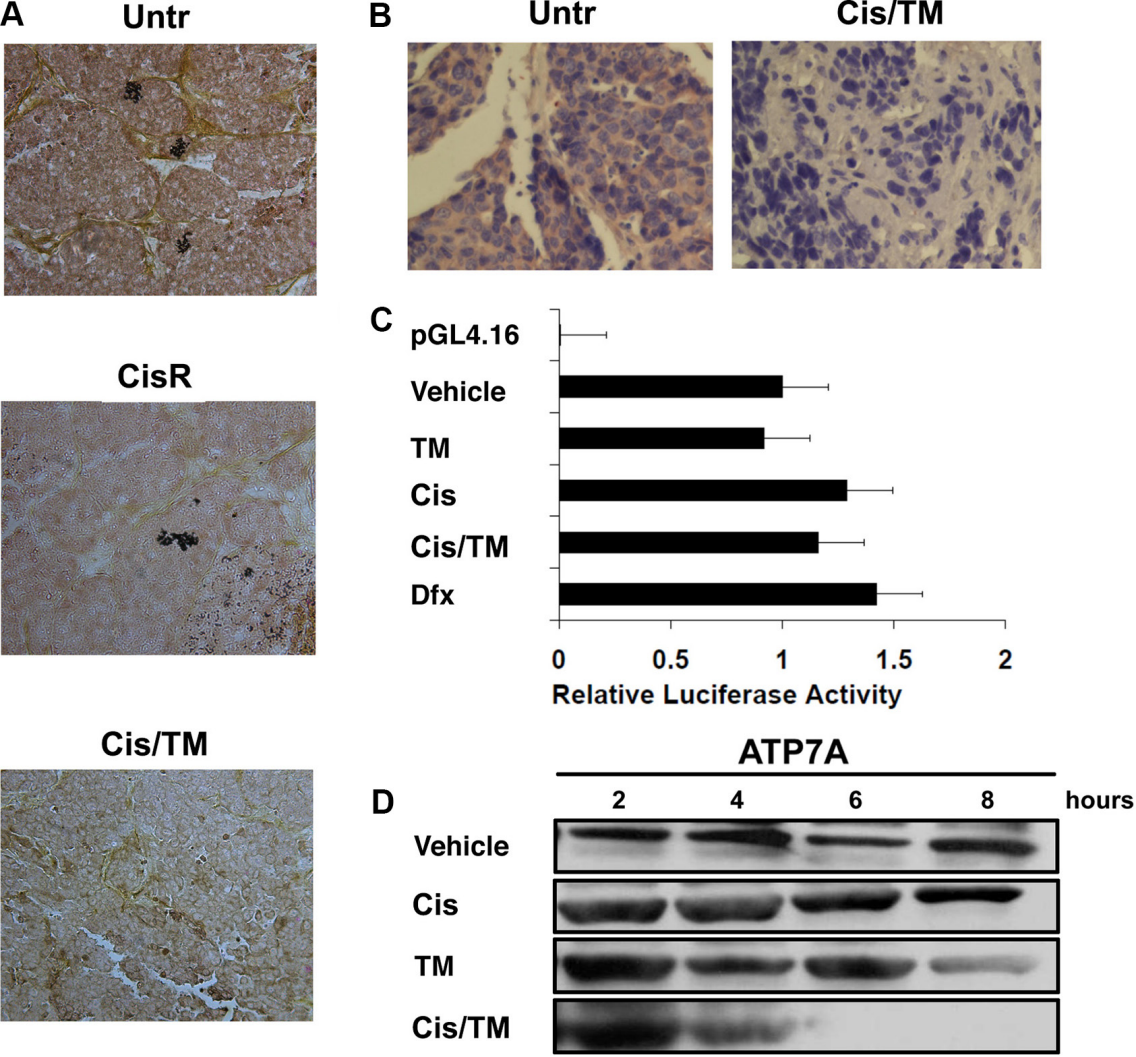
Investigation of ATP7A downregulation by TM in cisplatin resistant breast cancer (**A**) TM treatment reduces copper level in cisplatin/TM (Cis/TM) treated cancers compared with untreated (Untr) and cisplatin mono-treated tumors (CisR). (**B**) Immunohistochemical staining of ATP7A in untreated and cisplatin/TM treated mouse allograft tumors showed that cisplatin/TM (Cis/TM) treated tumors had significantly less ATP7A than untreated tumors (Untr). (**C**) Relative luciferase activity assay demonstrated no significant change in ATP7A minimal promoter activity regardless of 24 hours treatment with vehicle, TM, cisplatin, cisplatin/TM, or the hypoxia-inducing Dfx. (**D**) Western blot against ATP7A in MDA-MB-231 cells under treatment with 36 μM cisplatin, and/or 10 μM TM.

TM was reported to reduce the copper transporter CTR1 in cervical and ovarian carcinoma [36, 37]. Thus, we hypothesized that TM may also decrease ATP7A protein expression level through copper chelation. Copper staining of allograft tumor sections showed high copper levels in both untreated (Untr) and cisplatin mono-treated resistant (CisR) mouse tumors, while cisplatin/TM double treated (Cis/TM) mouse tumors prevailed low copper levels (Figure 6A). In concert to lower copper levels, immunohistochemical staining of antibody against ATP7A on mouse tumor sections showed that cisplatin/TM double treatment (Cis/TM) reduced ATP7A protein levels as compared to untreated control (Untr) (Figure 6B).

We next asked whether ATP7A protein downregulation is due to inhibition of *ATP7A*'s gene transcription. Relative luciferase activity of ATP7A -224 HIF2 promoter was tested with cisplatin addition in the presence or absence of TM in cells with no significant changes in promoter activity in TM treated (TM) group as compared to negative control (Vehicle), whereas cisplatin (Cis), cisplatin/TM (Cis/TM) and hypoxia-inducing desferrioxamine mesylate (Dfx) treated groups showed 1.3, 1.2 and 1.4 fold increase in *ATP7A* promoter activity respectively (Figure 6C). This result suggested that TM alone or in combination with cisplatin does not significantly induce or inhibit *ATP7A*'s promoter activity.

In contrast to *ATP7A*'s promoter activity, *in vitro* treatment of cell cultures with drugs showed that addition of TM (TM) slightly reduced ATP7A protein levels 8 hours post-treatment, whereas cisplatin/TM double treatment (Cis/TM) led to ATP7A protein downregulation 6 hours post-treatment; alternatively, cisplatin treatment alone (Cis) resulted in no changes in ATP7A protein levels as compared to vehicle control (Vehicle) (Figure 6D). Therefore, this data suggested that ATP7A downregulation upon cisplatin/TM double treatment occurred at protein level. Taken together, TM reduces ATP7A protein level *in vitro* and *in vivo*, without significant effect on *ATP7A* gene transcription.

Collectively, this data supports a model that increased ATP7A sequestering of cisplatin contributes to resistance by preventing it from reaching the nucleus, and TM treatment, which effectively reduces ATP7A, may reverse this process, leading to enhanced nuclear localization of cisplatin and consequently, DNA damage and apoptosis.

## DISCUSSION

The development of drug resistance presents a major impediment to the treatment of breast cancer [7, 38, 39]. *BRCA1-*mutant breast cancers respond well to platinum agent therapy, which induces DNA crosslinking, as the tumors are deficient in DNA repair; however, cisplatin resistance often develops [12, 13, 40–43]. Platinum resistance was caused by numerous reasons, mainly covering the influx and efflux of platinum, and overcome of cytotoxicity mechanisms [9, 15, 16].

In this study, we focused on elucidation of the specific biological roles of ATP7A in cisplatin resistant breast cancer. We observed a significant increase in drug efficacy in platinum resistant breast cancer cells when we combined RNAi directed at *ATP7A* with cisplatin. In an effort to target the copper transport pathway pharmacologically, we found treatment of cells with TM could achieve comparable cytotoxicity yielded from *ATP7A* knockdown. TM is an active copper chelating agent used to treat disorders of copper metabolism, such as Wilson's disease, and serves as an anti-angiogenesis agent [28, 31]. It has been shown that TM-copper chelation has a good therapeutic effect for a number of solid tumors [34, 35].

It was previously shown that TM could increase cisplatin sensitivity and efficacy in cervical and ovarian cancer cells in a CTR-1 dependent manner [36]. However, our study revealed additional effects of TM on transport downstream of CTR-1 and the exact contribution of ATP7A, a protein attributed to platinum efflux and implicated in platinum sequestering, to platinum resistance [44–46]. We demonstrated that the ability of TM to increase tumor sensitivity to cisplatin primarily through effects on ATP7A, i.e. by reducing the amount of ATP7A protein in cisplatin resistant tumor cells. We suspected that DNA damage was caused by increased nuclear localization of cisplatin. The reduction in ATP7A was also associated with reduced colocalization between ATP7A and labeled platinum, and increased colocalization between nuclear DNA and labeled platinum, suggesting that lower levels of ATP7A resulted in less sequestering of platinum.

We have previously shown that prolonged treatment with cisplatin triggers accumulation of cisplatin resistant CSCs [13]. We now found that the combination of TM and cisplatin could prevent accumulation of CSCs, accompanied by widespread DNA damage and apoptosis. To uncover the mechanism underlying the synergistic actions between cisplatin and TM, we performed microarray analysis. It revealed that combined treatment of cisplatin and TM induced changes both in gene expression level and the number of genes that are involved in DNA damage repair (DDR), cell cycle checkpoint and apoptosis at much higher frequency than the treatment of either drug alone. This finding provides the molecular basis for the synergy between these two drugs.

**Figure 7:**
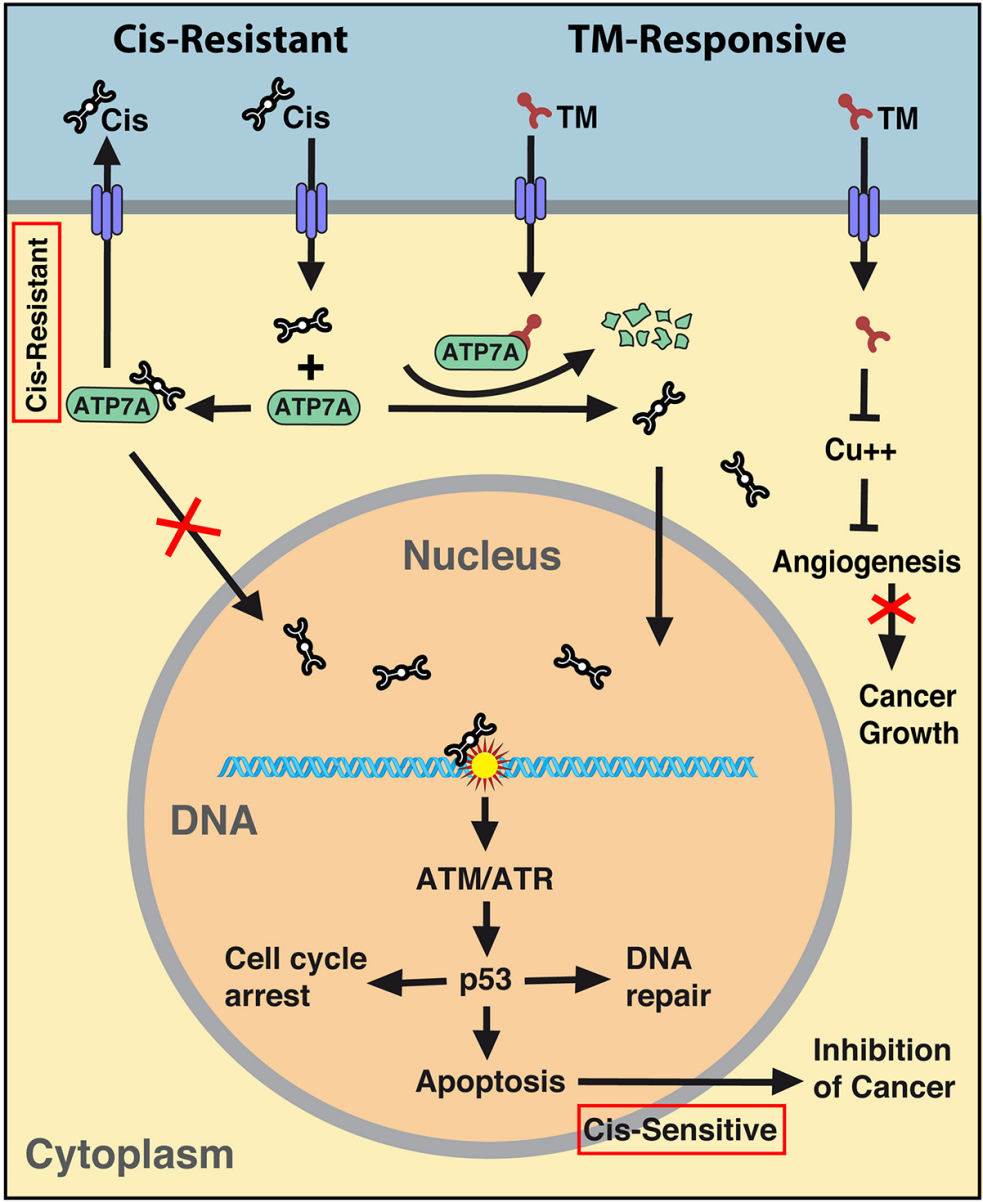
Summary of the mechanism regarding synergy between cisplatin and TM Cisplatin resistant tumors express increased levels of ATP7A that sequester cisplatin in the cytoplasm to prevent its nuclear localization and also pump cisplatin out of cells. The presence of TM reduces intracellular ATP7A levels, facilitates nuclear localization of cisplatin, enhances DNA damage, hence sensitizes cisplatin resistant tumor to cisplatin therapy.

In summary, our data revealed that ATP7A plays a critical role in creating platinum resistance through sequestering cisplatin in the cytoplasm and pumping it out of the cell. TM treatment reduces ATP7A concentration and facilitates cisplatin localization to the nucleus in order to trigger DNA damage. This, in turn, triggers activation of DNA damage response involving ATM/ATR signaling that activates p53. The activation of p53 activates multiple cell cycle checkpoints and triggers cell cycle arrest, which allows cells to repair damaged DNA. Meanwhile, p53 also triggers apoptosis if the damage is too extensive to be repaired, resulting in reduced cell proliferation and inhibition of cancer growth (Figure 7). As cisplatin is widely used for cancer therapy and cisplatin resistance is a common problem associated with chemotherapy, our finding provides a clue to combat platinum resistant tumors and sensitize patients to conventional breast cancer treatment strategies.

## MATERIALS AND METHODS

### Human RNAi candidate screen

A small-scale screen of a Thermo Dharmacon human custom siRNA siGENOME library comprised of 55 hand-selected breast cancer chemotherapy resistance-related gene targets was carried out *in vitro* (Supplementary Table S1). These genes were identified in the common genomic gain regions found in the cisplatin resistant breast cancer cells and associated with poor-prognosis breast cancer, including 6p12, 6p21, 11q13, 20q13.2 and several regions of 14q [18–21]. Additionally, we added siRNA targeting genes related to stem cell maintenance to the candidate screen, such as *SOX2* and *OCT3/4*, predicting that a small cancer stem cell population maintained or evolved in cisplatin treated tumors could drive or contribute to resistance. Finally, we included several siRNAs to knock down drug detoxifying enzymes as well as drug and metal transporters.

This screen utilized several human breast cancer cell lines that were determined to be resistant to cisplatin by the National Cancer Institute (NCI) *In Vitro* Cell Line Screening Project (IVCLSP) (http://dtp.nci.nih.gov/branches/btb/ivclsp.html).

### Cell transfection

For RNAi screen, T47D, MCF7 and MDA-MB-231 human breast cancer cells (ATCC) were transfected with 55 distinct siRNA pools using Dharmfect4 at 25 nM in 96-well plates seeded with 5,000 cells/well in triplicate. RNAi transfections were performed both with and without cisplatin treatment at 10 μM for MCF7 and 36 μM for MDA-MB-231 and T47D, and knockdown was validated by Western blot and quantitative RT-PCR with a consistent 88–92% knockdown efficiency achieved in three distinct human breast cancer cell lines (Supplementary Figure S1). Cells were analyzed for ATP7A expression by quantitative RT-PCR using the following primers: 5′ GCTCCTATCCAGCAGTTTGC 3′ and 5′ ACAGGGACATGCGATACACA 3′. Sensitivity to cisplatin was assayed by ATP release using the Promega Cell Titer Non-Radioactive Cell Proliferation Assay Kit and/or the 3-(4,5-dimethylthiazol-2-yl)-2,5-diphehyl-2H-tetrazolium bromide (MTT) assay. Lamin positive controls and a non-target control were run with each assay.

For pharmacological replacement, 5,000 cells/well of the indicated cell lines was seeded into a 96-well plate in triplicate. 16 hours cultures were added with gradient concentrations of ammonium tetrathiomolybdate (TM) (Sigma-Aldrich, cat# 323446) as indicated in corresponding figures in the absence or presence of 10 μM cisplatin (Sigma-Aldrich, cat# P4394). Cells were incubated at 37°C at 5% CO_2_ for 72 hours after drug addition. MTT assay was performed as instructed by manufacturer's protocol (Roche Diagnostics cat. 11465007001). All raw data was normalized to DMSO control. Predicted addictive curve was calculated by multiplying normalized mean cell viability of TM mono-treatment by mean cell viability of cisplatin mono-treatment used in this study, which is 70%. For example for doses of TM that generate 80% and 50% viability, respectively, the addictive point is 56% (70% × 80%), and 35% (70% × 50%), respectively. Graphs were plotted using GraphPad Prism 6.

### Allograft and xenograft mouse models

Adherence to the NIH Guide for the Care and Use of Laboratory Animals was followed for all *in vivo* experiments. All allograft and xenograft *in vivo* experiments followed protocols described earlier [13]. Briefly, 6–10 weeks old female aythymic nude mice were implanted with *BRCA1* mutant breast cancer cells in the bilateral 4^th^ mammary fat pads. 1 × 10^6^ cells were implanted subcutaneously and tumors became visible 7–14 days post-implantation. TM and cisplatin treatment was initiated when tumors reached 200 mm^3^. Mice were intraperitoneally injected with cisplatin at a dose of 6 mg/kg body weight twice per week with or without TM treatment at 0.015–0.030 mg/ml continuously in drinking water. Tumor volume was measured 2–4 times per week and compared between different treatment groups of mice (N = 10–12 tumors per group). Tumor volume was calculated using the formula: *V* = *ab*^2^/2, where a and b is tumor length and width, respectively. *In vivo* experiments were performed in triplicate with 5–6 mice per treatment group.

### Isolation and staining of mouse tumor cells for imagestream analysis

Mice were euthanized by CO_2_ inhalation and tumors excised to create single cell suspensions. Tumor tissue was finely minced under sterile conditions in DMEM (Cellgro Mediatech) containing 10% FBS, 1% glutamine, 10 ng/mL epidermal growth factor (EGF) (Invitrogen), and 5 μg/mL insulin (Sigma-Aldrich), and passed through a 45 micron nylon cell strainer (BD Falcon) to create single cell suspension. Cells were pelleted and plated on 100 mm polystyrene plates (Corning) at a concentration of 3 × 10^6^ cells per plate in 5 mL of DMEM media described above. Cells were left undisturbed and allowed to attach in a 37°C incubator with 5 percent CO^2^ for 3 days and then passaged or frozen in liquid nitrogen. 1 × 106 live tumor cells at passage 2 or younger were incubated for 1–2 hours on ice in dark with cisplatin labelled with Kreatech Platinum Bright 570 Red/Orange Reagent (Kreatech/BioMicroSystems) in PSS buffer containing phosphate buffered saline pH 7.2, 0.5% bovine serum albumin, and 2 mM ethylenediaminetetraacetic acid (EDTA). The labeled cisplatin is comprised of mono-reactive cisplatin derivatives that react at the N7 positions of guanine moieties in DNA. After incubation, cells were washed again in PSS buffer and fixed in 2% paraformaldehyde and stained with anti-ATP7A primary antibody produced in chicken (Sigma-Aldrich) for 30–60 minutes on ice in dark, then an anti-chicken Alexa488 secondary antibody (Molecular Probes) for 30 minutes on ice in dark and nuclei were stained with 4′,6-diamidino-2-phenylindole (DAPI).

### Imaging flow cytometry

Localization and similarity analyses were performed on Amnis ImageStream X multi-spectral imaging flow cytometer. Alexa488-labeled ATP7A and fluorophore-labeled cisplatin were excited by a 488nm laser and collected in channels containing 470–560 nm and 560–595 nm filters, respectively. DAPI was excited by a 405 nm laser and collected in the channel containing 430–505 nm filter. 5000–10,000 cells were imaged and data were compensated and analyzed using Amnis IDEAS software. The degree of co-localization between ATP7A and cisplatin (Pt), ATP7A and DAPI, and cisplatin (Pt) and DAPI was assessed using the Similarity Feature included in the software package, based on single, focused cells. The Similarity Feature is a measure of the degree to which two input images within a masked region are linearly correlated and is a pixel by pixel comparison based on the log transformed Pearson's coefficient. The ImageStream analyses were performed in triplicate utilizing cell populations from at least 3 tumors for each treatment group.

### Microarray analysis

1.0 × 10^5^ basal type MDA-MB-231 breast cancer cells (ATCC) were treated with 36 μM cisplatin (Sigma), 10 μM TM, both drugs, or a vehicle control in triplicate in 6-well plates and harvested at 12 and 24 hours. RNA was prepared from cell pellets using the Qiagen RNeasy RNA purification kit. RNA quality was determined by 2100 Bioanalyzer analysis (Agilent Technologies) and microarray was performed in triplicate for each drug treatment group using Affymetrix gene Chip Human Gene 1.0 ST Arrays (901086). ANOVA and pathway analysis was performed by the NIDDK Genomic Core Facility. The microarray data have been submitted to the GEO database under the accession number GSE77515.

### Statistical analysis

Raw intensity data were normalized by the Robust Multi-array Average (RMA) method [47] using the ‘affy’ package in R-Bioconductor. Normalized data were performed in R–Bioconductor using ‘limma’ package to identify differentially expressed genes between different treatment samples and control samples at each time point [48]. The list of differentially expressed genes at each time point of each treatment group was further filtered by the criteria of *P <* 0.05 and fold change > 1.5 to identify the set of significant differentially expressed genes in each group. Gene function enrichment was analyzed using analysis of variance (ANOVA) and Fisher's exact test. Unless otherwise stated, *P* values were considered significant if *P* < 0.05. Error bars represent SEM of 3 experiments unless otherwise stated. All statistical analysis was performed using R Statistical Software (version 3.1.2; R Foundation for Statistical Computing, Vienna, Austria).

## SUPPLEMENTARY MATERIALS FIGURES AND TABLES




