# Supplementary material for: Ammonium tetrathiomolybdate treatment targets the copper transporter ATP7A and enhances sensitivity of breast cancer to cisplatin
Source: Oncotarget. 2016 Oct 31;7(51):84439–52. doi: 10.18632/oncotarget.12992 (PMC5341295; doi:10.18632/oncotarget.12992)
Supplement: Supplementary file 1 [file oncotarget-07-84439-s001.pdf]

Ammonium tetrathiomolybdate treatment targets the copper transporter ATP7A and enhances sensitivity of breast cancer to cisplatin

Supplementary Materials

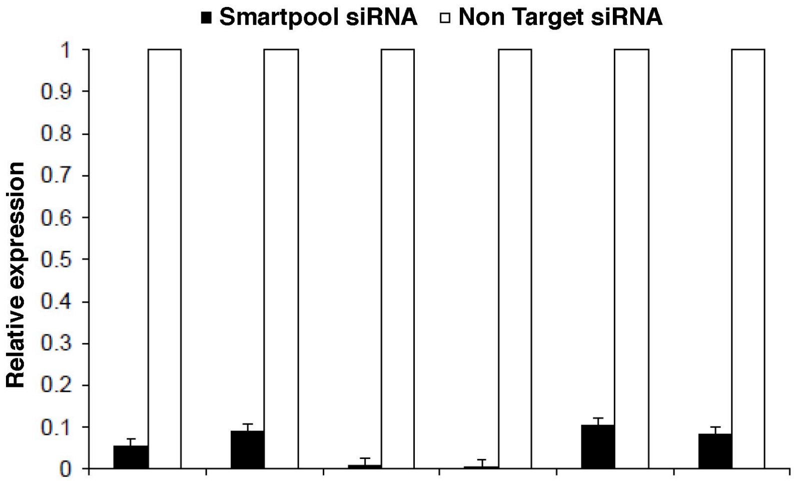

	Mouse breast tumor line 50 nM Lamin	Cisplatin-R mouse breast tumor line 50 nM Lamin	T47D 100 nM Lamin	T47D 50 nM Lamin	T47D 20 nM Lamin	T47D 100 nM ATP7A
Smartpool siRNA	0.056133	0.0889165	0.007	0.00347	0.1047	0.08171
Non Target siRNA	1	1	1	1	1	1

Supplementary Figure S1: Validation of siRNA knockdown in both mouse and human breast cancer cells. Various amount of siRNA for lamin, *ATP7A* or non-target siRNA were transfected into mouse mammary cancer cell 69 (either parental or cisplatin resistant derivative), or T47D human breast caner cells. Knockdown was visualized by RT-qPCR and the values of non-target siRNA were set as 1. Efficiency of *ATP7A* knockdown was also analyzed by RT-qPCR using the following primers: 5′ GCTCCTATCCAGCAGTTTGC 3′ and 5′ ACAGGGACATGCGATACACA 3′.

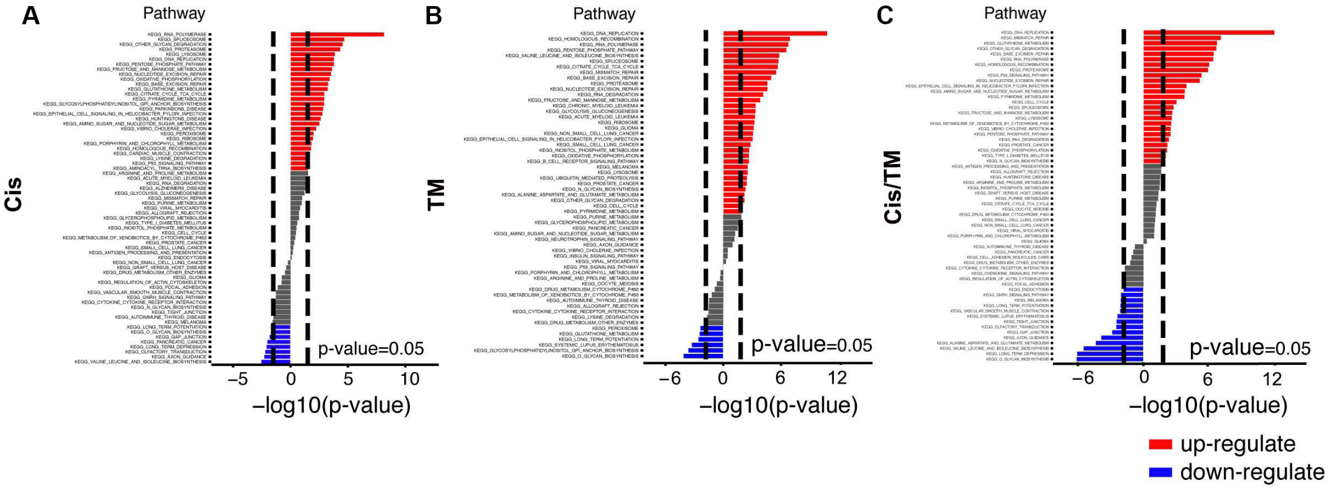

Supplementary Figure S2: Pathway analysis of common genes obtained from microarray analysis of MDA-MB-231 cells treated with (A) cisplatin, (B) TM, and (C) cisplatin/TM corresponding to Figure 4A–4C. Enrichment score of upregulated genes was expressed as its –log10(p-value) (red bars) while enrichment score of downregulated genes was expressed as its log10(p-value) (blue bars). The dotted line indicates the cutoff threshold of p-value = 0.05.

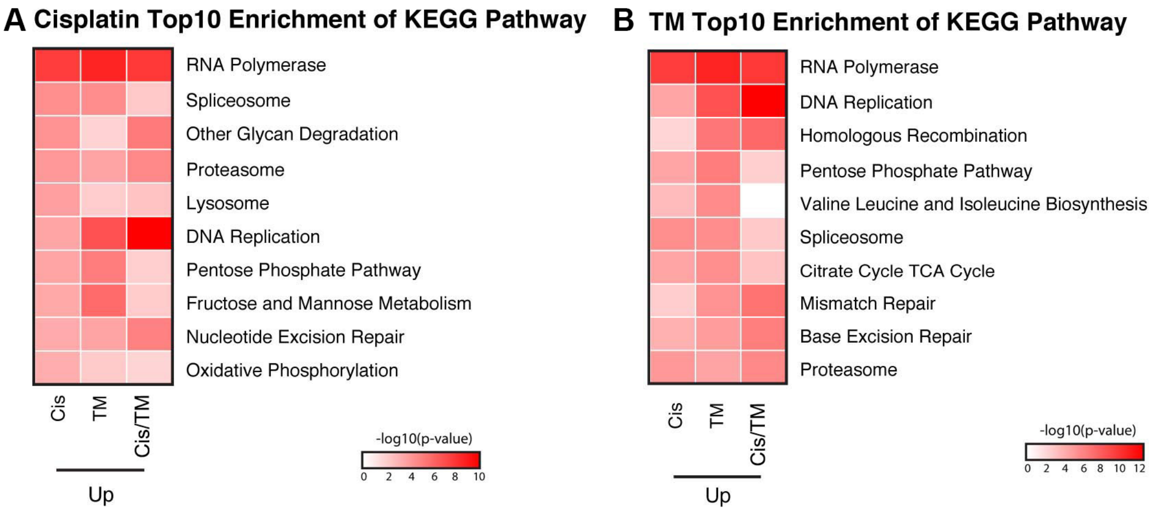

Supplementary Figure S3: KEGG pathway enrichment heatmap to demonstrate top 10 upregulated pathways in (A) cisplatin and (B) TM mono-treated cells. Top 10 upregulated pathways of the indicated treatment groups were sorted out, corresponding changes in total number of upregulated genes (common genes taken from overlapping area in Venn diagram in Figure 4A–4B) were shown.

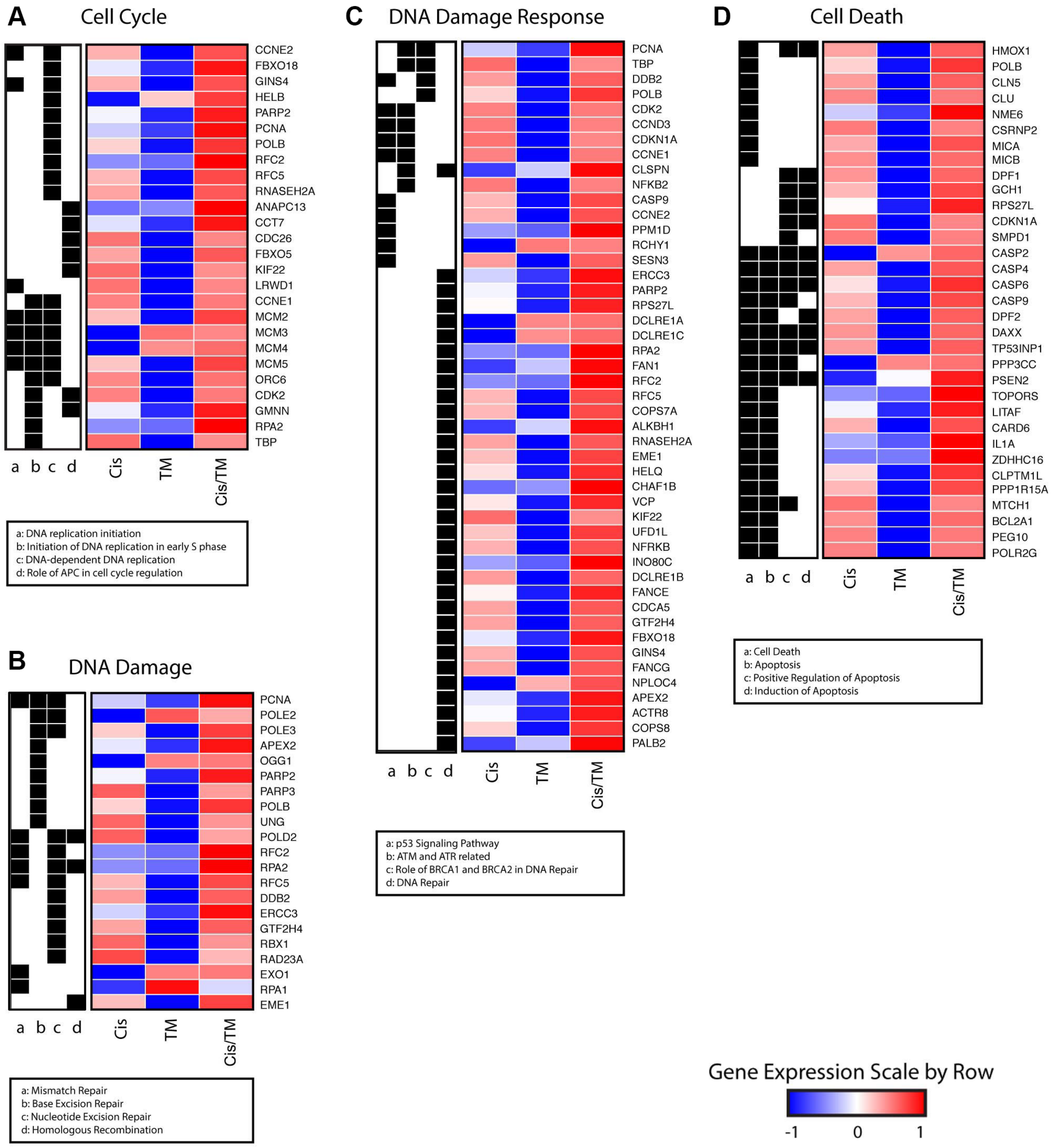

Supplementary Figure S4: Heatmap of individual genes' expression classified according to the pathways of (A) cell cycle, (B) DNA damage, (C) DNA damage response, and (D) cell death. Color bars reflect levels of gene expression and black bars indicate their involvement in different sub-pathways classified as A, B, C, and D shown in the boxes under each panel.

Supplementary Table S1: Genes selected from human genomic gain regions common in cisplatin resistant breast cancer lines

Genomic Region	Genes of Interest	Cisplatin or Multidrug Resistance Related	BRCA1/DNA Repair Related	Stem Cell Related
**3q26.2**	SOX2			SOX2
**6p12**	GSTA1, GSTA 2, IL17A, CENPQ, TNFRSF21	GSTA1/2 (cis) IL17A (tam) CENPQ (irin) TNFRSF21 (mdr)	CENPQ	
**6p21**	OCT3/4, HMGA1, FANCE, SRPK1	HMGA1 (cis) FANCE (cis) SRPK1 (cis)	HMGA1, FANCE	OCT 3/4
**7q35**	hERG	hERG (cis)		
**11q13**	TIP60, REL-A (p65), ARL2, MUS81, BAD, EMS1, UCP2, UCP3, CCS, MEN-1, CDCA5, GSTp1,	TIP60 (cis) REL-A (cis) ARL2 (mdr) BAD (cis) EMS1 UCP2/3(mdr) CCS (cis)	TIP60 (BRCA1 Independent) REL-A (binds BRCA1) MUS81, MEN-1, CDCA5	TIP60
**11q14**		unknown		
**14q11**	PARP2, NDRG2, PSMB5, hnRNPC,	PARP2(cis) NDRG2(cis) PSMB5 hnRNPC (cis, act)	PARP2, APEX1	
**14q12**	G2E3		G2E3	
**14q32.33**	XRCC-3 (RAD51-like)		XRRC-3	
**17q23.2**	BRIP1, ABC1	ABC1(mdr)	BRIP1	
**20q13.2**	BCAS1, AURKA	BCAS1(tam)	AURKA	
**Other genes**	HMGA2, CSNK2A1, CCR7, BRCA2, HMGN2, MDR1, MKP1, MKP2, STC1, PXR, FXR, PARP1, ELF-1, OCT 6, TXN, RRM1, STAT3, NOS-2, EMSY, CXCR4, FANCG, CSNK2A2			

Abbreviations: tam = tamoxifen; cis = cisplatin; mdr = multi drug; act = actinomycin; irin=irinotecan.

Supplementary Table S2: Sequences for human candidiate cisplatin resistance rnai library.

See Supplementary_Table_S2

Supplementary Table S3: Number of genes showed expression change induced by cisplatin and/or TM in MDA-MB-231 cells with both *p* < 0.05 and fold change of ≥ 1.5 using ANOVA analysis

Sample	Treatment	No. of total genes	No of up-regulated genes	No. of down-regulated genes
1	Cisplatin 12H	5884	3710	2174
2	Cisplatin 24H	4127	3035	1092
3	TM 12H	3539	2711	828
4	TM 24H	2119	1530	589
5	Double 12H	5665	3833	1832
6	Double 24H	2088	1491	597
